# Meta-analyses of chemotherapy for locally advanced and metastatic pancreatic cancer: results of secondary end points analyses

**DOI:** 10.1038/sj.bjc.6604436

**Published:** 2008-06-24

**Authors:** A Sultana, C Tudur Smith, D Cunningham, N Starling, J P Neoptolemos, P Ghaneh

**Affiliations:** 1 CRUK Liverpool Cancer Trials Unit, Cancer Research Centre, 200 London Road, Liverpool, L3 9TA, UK; 2 Centre for Medical Statistics and Health Evaluation, University of Liverpool, Shelley's Cottage, Brownlow Street, Liverpool L69 3GS, UK; 3 Department of Medicine, Royal Marsden Hospital, Downs Road, Sutton, Surrey SM2 5PT, UK

**Keywords:** meta-analyses, pancreatic cancer, chemotherapy

## Abstract

In advanced pancreatic cancer, level one evidence has established a significant survival advantage with chemotherapy, compared to best supportive care. The treatment-associated toxicity needs to be evaluated. This study examines the secondary outcome measures for chemotherapy in advanced pancreatic cancer using meta-analyses. A systematic review was undertaken employing Cochrane methodology, with search of databases, conference proceedings and trial registers. The secondary end points were progression-free survival (PFS)/time to progression (TTP) (summarised using the hazard ratio (HR)), response rate and toxicity (summarised using relative risk). There was no significant advantage of 5FU combinations *vs* 5FU alone for TTP (HR=1.02; 95% CI=0.85–1.23) and toxicity. Progression-free survival (HR 0.78; CI 0.70–0.88), TTP (HR=0.85; 95% CI=0.72–0.99) and overall response rate (RR=0.56; 95% CI=0.46–0.68) were significantly better for gemcitabine combination chemotherapy, but offset by the greater grade 3/4 toxicity thrombocytopenia (RR=1.94; 95% CI=1.32–2.84), leucopenia (RR=1.46; 95% CI=1.15–1.86), neutropenia (RR=1.48; 95% CI=1.07–2.05), nausea (RR=1.77; 95% CI=1.37–2.29), vomiting (RR=1.64; 95% CI=1.24–2.16) and diarrhoea (RR=2.73; 95% CI=1.87–3.98). There is no significant advantage on secondary end point analyses for administering 5FU in combination over 5FU alone. There is improved PFS/TTP and response rate, with gemcitabine-based combinations, although this comes with greater toxicity.

Advanced pancreatic cancer has a poor prognosis, with a median survival of 2–6 months for metastatic disease and 6–11 months for locally advanced disease (Cancer Research, 2006). Chemotherapy with fluoropyrimidines, gemcitabine, either alone or in combination with other agents (Rocha Lima and Flores, 2006), and chemoradiation are all used in the palliative setting (Mancuso *et al*, 2006). Overall survival meta-analyses, using relative risk (Yip *et al*, 2006) or the hazard ratio (HR) (Fung *et al*, 2003; Sultana *et al*, 2007), have established a role for chemotherapy over best supportive care. Questions have arisen as to the cost at which this survival advantage is gained, in particular, the toxicity profile. Following from our previous survival meta-analysis (Sultana *et al*, 2007), we present the results of the secondary outcome measures meta-analysis.

There has only been one fully published meta-analysis evaluating secondary outcome measures, with no pooling of the results of these end points (Yip *et al*, 2006). Other published reports have assessed this only for the comparison of gemcitabine combinations *vs* gemcitabine. (Liang, 2005; Milella *et al*, 2006; Heinemann *et al*, 2006a; Xie *et al*, 2006a, 2006b; Bria *et al*, 2007; Heinemann *et al*, 2007). To fully evaluate the risks *vs* the benefits of treatment, a comprehensive evaluation including assessment of several composite end points is required.

## Methods

Detailed description of the methodology of the systematic review has already been described (Sultana *et al*, 2007).

The secondary outcome measures evaluated were progression-free survival (PFS – time from randomisation to progression or death) or time to progression (TTP – time from randomisation to disease progression), overall response rate (ORR – number of partial and complete responses) and toxicity (as published by the trialists, was recorded, with the most frequently reported events analysed).

Individual trial level time to event data (PFS/TTP) were summarised by the log HR and its variance was approximated using previously reported methods (Parmar *et al*, 1998; Williamson *et al*, 2002). Trial level log HRs and their variances were pooled using an inverse variance, weighted average and results presented as a HR and 95% confidence interval.

Dichotomous data (ORR and toxicity) were summarised using relative risks and 95% confidence intervals and pooled using the Mantel–Haenszel method for combining trials (Deeks *et al*, 2001). Heterogeneity was assessed by visual inspection of the Forrest plot, the Cochran's *χ*^2^ test (using a 10% significance level, in view of the low power of tests for heterogeneity (Paul and Donner, 1992)) and interpretation of the I^2^ statistic (percentage of variation due to heterogeneity with higher values indicating a greater degree of heterogeneity) (Deeks *et al*, 2004). A fixed effect approach was adopted unless there was evidence of significant unexplained heterogeneity in which case a random effects approach was used.

## Results

Results are presented for the comparisons with adequate data to assess the secondary outcome measures.

### 5FU *vs* 5FU combination chemotherapy

There were five studies (Supplementary Table 1) (Kovach *et al*, 1974; Cullinan *et al*, 1985, 1990; Ducreux *et al*, 2002; Maisey *et al*, 2002) (*n*=700) included in this comparison. A HR of <1 indicates a survival advantage for 5FU combination chemotherapy.

**Figure 1:**
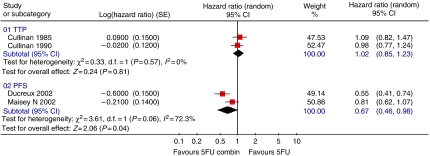
5FU single agent *vs* 5FU-based combination chemotherapy – PFS/TTP analyses.

**Figure 2:**
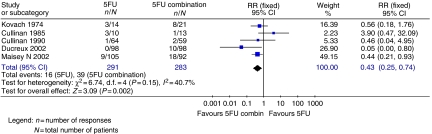
5FU single agent *vs* 5FU-based combination chemotherapy – response rate analyses.

**Figure 3:**
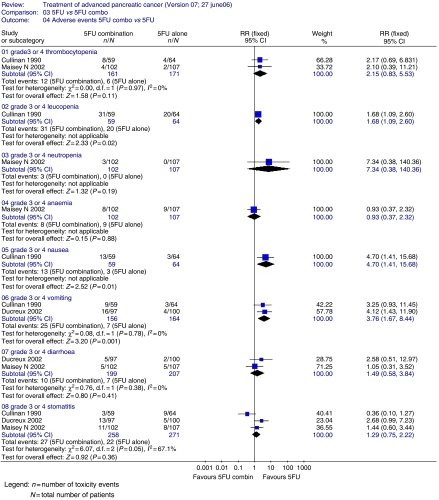
5FU single agent *vs* 5FU-based combination chemotherapy – toxicity analyses.

Two trials assessed TTP (Figure 1) and found no significant advantage for 5FU combinations over 5FU alone (HR=1.02; 95% CI=0.85–1.23). For PFS, 5FU combination appeared better than 5FU alone (two trials; 416 patients; HR=0.67; 95% CI=0.46–0.98). The ORR (Figure 2) was superior (five trials; 700 patients; RR=0.43; 95% CI=0.25–0.74) in the 5FU combination arm. Grade 3 or 4 vomiting was significantly greater in the 5FU combination chemotherapy arm (two trials; 320 patients; RR=3.76; 95% CI=1.67–8.44). There was a higher occurrence of diarrhoea (two trials 406 patients; RR=1.49; 95% CI=0.58–3.84), stomatitis (three trials; 529 patients; RR=1.29; 95% CI=0.75–2.22) and thrombocytopenia (two trials; 332 patients; RR=2.15; 95% CI=0.83–5.53) in the combination chemotherapy arm (Figure 3). Data for leucopenia, neutropenia, anaemia and nausea are displayed in Figure 3. There was significant between trial heterogeneity in the PFS analysis, unlike for the TTP and response rate analyses.

### Gemcitabine *vs* 5FU

Two randomised controlled trials involving 197 patients were assessed (Burris *et al*, 1997; Cantore *et al*, 2004), including unpublished individual patient data (Cantore *et al*, 2004). A HR of <1 indicates a survival advantage for gemcitabine. Gemcitabine resulted in survival advantage on TTP analysis, (HR=0.46; 95% CI=0.31–0.70), but not for PFS analysis (HR=0.94; 95% CI=0.58–1.53).

Overall response rate appeared better in the gemcitabine arm; however, the wide confidence interval suggests a benefit for either gemcitabine or 5FU (one trial; 126 patients; RR=0.14; 95% CI=0.01–2.66). In the Burris trial (Burris *et al*, 1997), haematological toxicity was seen more frequently following gemcitabine therapy (grades 3 and 4 neutropenia in 25% of gemcitabine and 4.9% of 5FU patients; *P*<0.001).

### Gemcitabine *vs* gemcitabine-based combination chemotherapy

Nineteen studies involving 4697 patients were included (Supplementary Table 2) (Berlin *et al*, 2002; Colucci *et al*, 2002; Wang *et al*, 2002; Heinemann *et al*, 2003; Scheithauer *et al*, 2003; Li and Chao, 2004; Ohkawa, 2004; Rocha Lima *et al*, 2004; Viret *et al*, 2004; Cunningham *et al*, 2005; Di Costanzo *et al*, 2005; Hermann *et al*, 2005; Louvet *et al*, 2005; Oettle *et al*, 2005; Reiss *et al*, 2005; Reni *et al*, 2005; Stathopoulos *et al*, 2005; Abou-Alfa *et al*, 2006; Poplin *et al*, 2006). Data from four of the included studies (Abou-Alfa *et al*, 2006; Heinemann *et al*, 2006b; Stathopoulos *et al*, 2006; Herrmann *et al*, 2007) were based on abstracts and extra data provided by the authors (Hermann *et al*, 2005; Stathopoulos *et al*, 2005). A HR of <1 indicates a survival advantage for gemcitabine-based combination chemotherapy.

**Figure 4:**
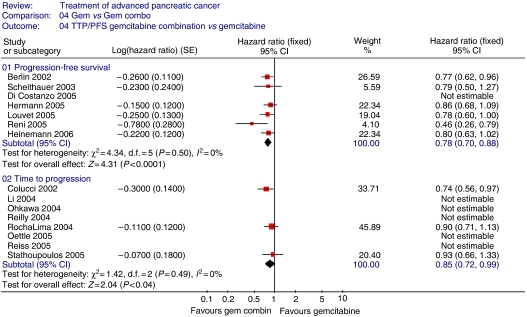
Results for gemcitabine *vs* gemcitabine-based combination chemotherapy – TTP/PFS.

**Figure 5:**
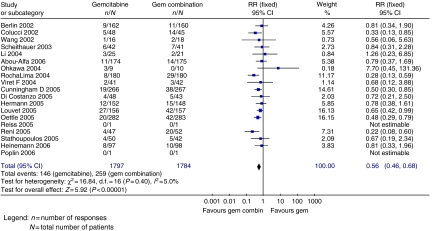
Results for gemcitabine *vs* gemcitabine-based combination chemotherapy – response rate.

**Figure 6:**
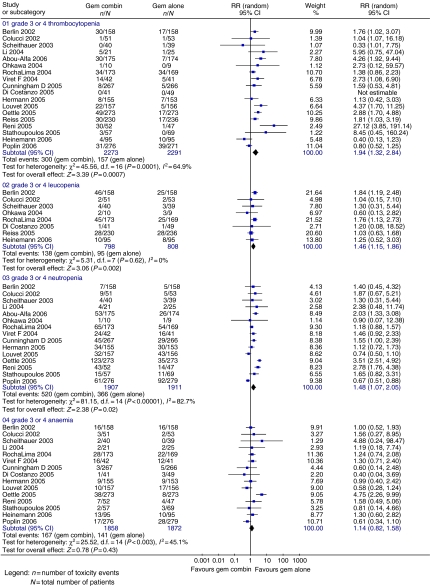
Results for gemcitabine *vs* gemcitabine-based combination chemotherapy – haematological toxicity.

**Figure 7:**
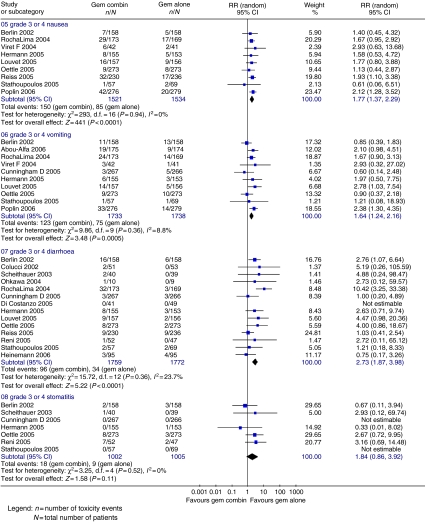
Results for gemcitabine *vs* gemcitabine-based combination chemotherapy – gastrointestinal toxicity.

Progression-free survival (four trials; 864 patients; HR=0.78; 95% CI=0.70–0.88), TTP (3 trials; 559 patients; HR=0.85; 95% CI=0.72–0.99) (Figure 4) and ORR (Figure 5) (17 trials; 3577 patients; RR=0.56; 95% CI=0.46–0.68) were significantly better in the gemcitabine combination chemotherapy arm. Haematological toxicity was greater in the gemcitabine combination chemotherapy arm (Figure 6), including thrombocytopenia (18 trials; 4564 patients; RR=1.94; 95% CI=1.32–2.84), leucopenia (eight trials; 1606 patients; RR=1.46; 95% CI=1.15–1.86), neutropenia (15 trials; 3818 patients; RR=1.48; 95% CI=1.07–2.05) and anaemia (15 trials; 3745 patients; RR=1.14; 95% CI=0.82–1.59). Gastrointestinal side effects (Figure 7) of nausea (nine trials; 3055 patients; RR=1.77; 95% CI=1.37–2.29), vomiting (10 trials; 3471 patients; RR=1.64; 95% CI=1.24–2.16) and diarrhoea (14 trials; 3531 patients; RR=2.73; 95% CI=1.87–3.98) were significantly increased, with a trend towards increased stomatitis (7 trials; 2007 patients; RR=1.84; 95% CI=0.86–3.92) in the gemcitabine combination chemotherapy arm. There was no significant inter-trial heterogeneity for the end points of PFS, TTP and ORR.

Examination of the funnel plots revealed evidence of bias, possibly publication bias, but this is difficult to interpret in view of the small number of studies within each comparison.

## Discussion

5FU combinations did not prolong TTP over 5FU alone, despite significantly better response rate with the former. The study of Yip *et al* (2006) assessed the parameters described in our analyses, but did not pool the results unlike our approach. In the two trials that had assessed PFS, the overall summary estimate favoured 5FU combination chemotherapy, but there was significant inter-trial heterogeneity. This may be due to the differences in dosing. The dose of 5FU administered was lower in the Maisey *et al* (2002) study (300 mg m^−2^ day^−1^ in both arms) compared to the Ducreux *et al* (2002) study (500 mg m^−2^ day^−1^ used in the single-agent arm and 1000 mg m^−2^ used in the combination arm).

As overall survival is a better indicator of efficacy than response rate (Maisey *et al*, 2002), the evidence from these end points, interpreted alongside the overall survival result (Sultana *et al*, 2007), do not support the use of 5FU combinations over 5FU single agent.

Meta-analyses of the secondary end points were not possible in the gemcitabine *vs* 5FU comparison, as these results were only available for one randomised trial.

Previous meta-analyses of secondary end points evaluating gemcitabine-based combinations *vs* gemcitabine employed differing survival analyses methodology (Liang, 2005; Heinemann *et al*, 2006a; Milella *et al*, 2006; Xie *et al*, 2006a). In contrast to these reports, our survival analyses were conducted using the HR, which is the ideal measure for time-to-event analyses, as it accounts for both censoring of data and the time it takes for the event (such as death or progression) to occur (Parmar *et al*, 1998).

For gemcitabine-based chemotherapy *vs* gemcitabine alone, our findings of improved PFS/TTP are in agreement with the meta-analyses of Xie *et al* (2006b). Better ORR with the combination regimens was in keeping with the studies of Xie *et al* and Milella *et al* (Xie *et al*, 2006b), while increased toxicity profile was noted by Xie *et al* (2006b). The meta-analyses that examined gemcitabine plus a platinum agent *vs* gemcitabine alone found better PFS/TTP in the combination arm (Xie *et al*, 2006a; Heinemann *et al*, 2007), significant improvement in ORR (Heinemann *et al*, 2007) and greater toxicity (Xie *et al*, 2006a).

We have done our utmost to cover most reported end points in the randomised controlled trials. We could not address quality of life due to the different methods used for reporting quality of life. Although we have pooled the response rate and adverse events data across studies to permit a clinically relevant analysis, reporting of these parameters varied. Response rates were reported using clinical parameters, the WHO and RECIST criteria, whereas the CTC, WHO and ECOG scales were used for toxicity data.

To conclude, there is insufficient evidence to suggest a TTP, response rate and toxicity advantage in administering 5FU in combination with other chemotherapy agents over 5FU alone. There is a small but significant TTP/PFS advantage, as well as improved response rate, with gemcitabine-based combinations, and this provides a justification for the use of these agents, despite their greater toxicity. An area for further randomised controlled trials to assess is which gemcitabine-based combination chemotherapy regimens are least toxic, while retaining all the other advantages of the combination approach.
