# Supplementary material for: Meta-analyses of chemotherapy for locally advanced and metastatic pancreatic cancer: results of secondary end points analyses
Source: Br J Cancer. 2008 Jun 24;99(1):6–13. doi: 10.1038/sj.bjc.6604436 (PMC2453014; doi:10.1038/sj.bjc.6604436)
Supplement: Supplementary Table 1 and 2 [file 6604436x1.doc]

Table 1: Included studies and outcome measures in the comparison of 5FU versus 5FU based combination chemotherapy

| **Trial** | **Group**  **(number randomised)** | **Stage of disease** | **Chemotherapy used** | **PFS**  **(hazard ratio)** | **TTP**  **(hazard ratio)** | **RR**  **(number of responses/total number of patients)** | **Toxicity- grade 3 or 4**  **(number of responses/total number of patients)** |
| --- | --- | --- | --- | --- | --- | --- | --- |
| Kovach  1974 | 5FU  (n=31) | na |  | not assessed | not assessed | 3/14 | na |
|  | Combination chemotherapy  (n=30) | na | plus BCNU |  |  | 8/21 | na |
| Cullinan  1990* | 5FU  (n=64) | na |  | not assessed | 0.98 | 1/64 | vomiting-3/64  nausea-3/64  stomatitis-9/64  leucopenia-20/64  thrombocytopenia-4/64 |
|  | Combination chemotherapy  (n=59) | na | FAP |  |  | 2/59 | vomiting-9/59  nausea-13/59  stomatitis-3/59  leucopenia-31/59  thrombocytopenia-8/59 |
| Cullinan  1985* | 5FU  (n=50) | Regional= 14  Metastatic=36 |  | not assessed | 1.09 | 3/10 | na |
|  | Combination chemotherapy  (n=50) | Regional= 15  Metastatic =35 | plus doxo and MMC |  |  | 1/13 | na |
| Ducreux  2002 | 5FU  (n=103) | Limited to pancreas= 8  Metastatic= 95 |  | 0.55 | not assessed | 0/98 | vomiting-4/100  diarrhoea-2/100  stomatitis-5/100 |
|  | Combination chemotherapy  (n=104) | Limited to pancreas= 13  Metastatic= 91 | plus CP |  |  | 10/98 | vomiting-16/97  diarrhoea-5/97  stomatitis-12/97 |
| Maisey N  2002 | 5FU  (n=107) | Locally adv= na  Metastatic= 70 |  | 0.81 | not assessed | 9/105 | diarrhoea-5/107  stomatitis-8/107  thrombocytopenia-2/107  neutropenia-0/107  anaemia-9/107 |
|  | Combination chemotherapy  (n=102) | Locally adv= na  Metastatic= 57 | plus MMC given as a protracted venous infusion |  |  | 18/92 | diarrhoea-5/102  stomatitis-11/102  thrombocytopenia-4/102  neutropenia-3/102  anaemia-8/102 |

Note: FA=5FU and Adriamycin; FAM= 5FU, adriamycin, mitomycin; BCNU= 1, 3 BIS-1- nitrosourea; CP= cisplatin; MMC= mitomycin; doxo=doxorubicin; Mallinson regime= 5FU, cyclophosphamide, methotrexate, vincristine; FAP= 5FU, doxorubicin, cisplatin; na= data not available; adv= advanced; panc ca= pancreatic cancer; yr= years; m= male; f= female

* 3 chemotherapy regimens were compared in the randomized controlled trials, but only two have been included for purposes of the meta-analyses, in order to avoid using the same set of data twice.

**Table 2: Included studies and outcome measures in the category of gemcitabine versus gemcitabine-based combination chemotherapy**

| **Trial** | **Group**  **(number randomised)** | **Stage of disease** | **Chemotherapy used** | **PFS**  **(hazard ratio)** | **TTP**  **(hazard ratio)** | **RR**  **(number of responses/total number of patients)** | **Toxicity- grade 3 or 4**  **(number of responses/total number of patients)** |
| --- | --- | --- | --- | --- | --- | --- | --- |
| Berlin  2002 | gem  (n=162) | Locally adv= 16  Metastatic= 146 |  | 0.77 | not assessed | 9/162 | thrombocytopenia-17/158  leucopenia-25/158  neutropenia-5/158  anaemia-16/158  nausea-5/158  vomiting-13/158  diarrhoea-6/158  stomatitis-3/158 |
|  | combo  (n=160) | Locally adv= 17  Metastatic= 143 | 5FU |  |  | 11/160 | thrombocytopenia-30/158  leucopenia-46/158  neutropenia-7/158  anaemia-16/158  nausea-7/158  vomiting-11/158  diarrhoea-16/158  stomatitis-2/158 |
| Colucci  2002 | gem  (n=54) | Stage II=11; III= 14; IV= 29 |  | not assessed | 0.74 | 5/48 | thrombocytopenia-1/53  leucopenia-2/53  neutropenia-5/53  anaemia-2/53  diarrhoea-0/53 |
|  | combo  (n=53) | Stage II=10; III= 10; IV= 33 | CP |  |  | 14/45 | thrombocytopenia-1/51  leucopenia-2/51  neutropenia-9/51  anaemia-2/51  diarrhoea-2/51 |
| Wang  2002 | gem  (n=20) | Stage III= 4; IV= 10 |  | na | na | 1/16 | na |
|  | combo  (n=22) | Stage III= 4; IV= 15 | CP |  |  | 2/18 | na |
| Heinemann  2006 | gem  (n=99) | na |  | 0.80 | not assessed | na | thrombocytopenia-10/95  leucopenia-8/95  anaemia-10/95  diarrhoea-4/95 |
|  | combo  (n=96) | na | CP |  |  | na | thrombocytopenia-4/95  leucopenia-10/95  anaemia-13/95  diarrhoea-3/95 |
| Scheithauer  2003 | gem  (n=42) | na |  | 0.79 | not assessed | 6/42 | thrombocytopenia-1/39  leucopenia-3/39  neutropenia-3/39  anaemia-0/39  diarrhoea-0/39  stomatitis-0/39 |
|  | combo  (n=41) | na | capecitabine |  |  | 7/41 | thrombocytopenia-0/40  leucopenia-4/40  neutropenia-4/40  anaemia-2/40  diarrhoea-2/40  stomatitis-1/40 |
| Li  2004 | gem  (n=25) | Metastases= 25 |  | not assessed | na | 3/25 | thrombocytopenia-1/25  neutropenia-2/25  anaemia-2/25 |
|  | combo  (n=21) | Metastases= 21 | CP |  |  | 2/21 | thrombocytopenia-5/21  neutropenia-4/21  anaemia-2/21 |
| Ohkawa  2004 | gem  (n=9) | Locally adv= 2  Metastatic= 7 |  | not assessed | na | 3/9 | thrombocytopenia-0/9  leucopenia-3/9  neutropenia-1/9  anaemia-0/39  diarrhoea-0/9 |
|  | combo  (n=10) | Locally adv= 3  Metastatic= 7 | UFT |  |  | 0/10 | thrombocytopenia-1/10  leucopenia-2/10  diarrhoea-1/10 |
| Oettle  2004 | gem  (n=282) | Stage II to III= 25; IV= 257 |  | not assessed | na | 20/282 | thrombocytopenia-17/273  neutropenia-35/273  anaemia-8/273  nausea-8/273  vomiting-10/273  diarrhoea-2/273  stomatitis-3/273 |
|  | combo  (n=283) | Stage II to III= 28; IV= 255 | pemetrexed |  |  | 42/283 | thrombocytopenia-49/273  neutropenia-123/273  anaemia-9/273  nausea-8/273  vomiting-9/273  diarrhoea-8/273  stomatitis-8/273 |
| O’Reilly  2004 | gem  (n=174) | na |  | not assessed | na | 11/174 | thrombocytopenia-7/174  neutropenia-26/174  vomiting-9/174 |
|  | combo  (n=175) | na | exatecan |  |  | 14/175 | thrombocytopenia-30/175  neutropenia-53/175  vomiting-19/175 |
| Rocha Lima  2004 | gem  (n=180) | Locally adv= 24  Metastatic= 145  Missing= 11 |  | not assessed | 0.90 | 8/180 | thrombocytopenia-24/169  leucopenia-25/169  neutropenia-54/169  anaemia-22/169  nausea-17/169  vomiting-14/169  diarrhoea-3/169 |
|  | combo  (n=180) | Locally adv= 27  Metastatic= 148  Missing= 5 | irinotecan |  |  | 29/180 | thrombocytopenia-34/173  leucopenia-45/173  neutropenia-65/173  anaemia-28/173  nausea-29/173  vomiting-24/173  diarrhoea-32/173 |
| Viret  2004 | gem  (n=41) | Stage III= 9  Stage IV= 32 |  | na | na | 2/41 | thrombocytopenia-5/41  neutropenia-16/41  anaemia-12/41  nausea-2/41  vomiting-1/41 |
|  | combo  (n=42) | Stage III= 8  Stage IV= 34 | CP |  |  | 3/42 | thrombocytopenia-14/42  neutropenia-24/42  anaemia-16/42  nausea-6/42  vomiting-3/42 |
| Hermann  2005 | gem  (n=159) | Locally adv= 21%  Metastatic= 79% |  | 0.86 | not assessed | 12/152 | thrombocytopenia-7/153  neutropenia-30/153  anaemia-9/153  nausea-5/153  vomiting-3/153  diarrhoea-3/153  stomatitis-1/153 |
|  | combo  (n=160) | Locally adv= 20%  Metastatic= 80% | capecitabine |  |  | 15/148 | thrombocytopenia-8/155  neutropenia-34/155  anaemia-9/155  nausea-8/155  vomiting-6/155  diarrhoea-8/155  stomatitis-0/155 |
| Stathopoulos  2005 | gem  (n=69) | na |  | not assessed | 0.93 | 4/50 | thrombocytopenia-0/69  neutropenia-11/69  anaemia-3/69  nausea-2/69  vomiting-1/69  diarrhoea-2/69  stomatitis-0/69 |
|  | combo  (n=57) | na | irinotecan |  |  | 5/42 | thrombocytopenia-3/57  neutropenia-15/57  anaemia-2/57  nausea-1/57  vomiting-1/57  diarrhoea-2/57  stomatitis-0/57 |
| Reni  2005 | gem  (n=47) | Locally adv= 14  Metastatic= 33 |  | 0.46 | not assessed | 4/47 | thrombocytopenia-1/47  neutropenia-14/47  anaemia-4/47  diarrhoea-0/47  stomatitis-2/47 |
|  | combo  (n=52) | Locally adv= 15  Metastatic= 37 | CP+epirub+5FU |  |  | 20/52 | thrombocytopenia-30/52  neutropenia-43/52  anaemia-7/52  diarrhoea-1/52  stomatitis-7/52 |
| Louvet  2005 | gem  (n=163) | Locally adv 30  Metastases 70 |  | 0.78 | not assessed | 27/156 | thrombocytopenia-5/156  neutropenia-43/156  anaemia-17/156  nausea-9/156  vomiting-5/156  diarrhoea-2/156 |
|  | combo  (n=163) | Locally adv 32  Metastases 68 | oxaliplatin |  |  | 42/157 | thrombocytopenia-22/157  neutropenia-32/157  anaemia-10/157  nausea-16/157  vomiting-14/157  diarrhoea-9/157 |
| Cunningham  2005 (interim analyses) | gem  (n=266) | Metastases 71% |  | na | na | 19/266 | thrombocytopenia-5/266  neutropenia-29/266  anaemia-5/266  vomiting-5/266  diarrhoea-3/266  stomatitis-0/266 |
|  | combo  (n=267) | Metastases 70% | capecitabine |  |  | 38/267 | thrombocytopenia-8/267  neutropenia-45/267  anaemia-3/267  vomiting-3/267  diarrhoea-3/267  stomatitis-0/267 |
| Reiss  2005 | gem  (n=236) | Metastatic= 77% |  | not assessed | na | na | thrombocytopenia-17/236  leucopenia-28/236  nausea-17/236  diarrhoea-9/236 |
|  | combo  (n=230) | Metastatic= 76% | 5FU+folinic acid |  |  | na | thrombocytopenia-30/230  leucopenia-28/230  nausea-32/230  diarrhoea-9/230 |
| DiCostanzo  2005 | gem  (n=48) | Locally adv= 13  Metastatic= 35 |  | na | not assessed | 4/48 | thrombocytopenia-0/49  leucopenia-1/49  anaemia-3/49  diarrhoea-0/49 |
|  | combo  (n=43) | Locally adv= 14  Metastatic= 29 | 5FU |  |  | 5/43 | thrombocytopenia-0/41  leucopenia-1/41  anaemia-1/41  diarrhoea-0/41 |
| Poplin 2006 | Gem  (n= 280) | na |  | na | na | na | thrombocytopenia-39/279  neutropenia-92/279  anaemia-28/279  nausea-20/279  vomiting-14/279 |
|  | Combo  (n=276) | na | oxaliplatin |  |  | na | thrombocytopenia-31/276  neutropenia-61/276  anaemia-17/276  nausea-42/276  vomiting-33/276 |

Note: CP=cisplatin; epirub=epirubicin; 5FU=5-Fluorouracil; gem = gemcitabine; na= data not available; gem= gemcitabine; combo= gemcitabine-based combination chemotherapy; adv= advanced; yr= years; m= male; f= female
